# Distribution and quantification of bioluminescence as an ecological trait in the deep sea benthos

**DOI:** 10.1038/s41598-019-50961-z

**Published:** 2019-10-10

**Authors:** Séverine Martini, Linda Kuhnz, Jérôme Mallefet, Steven H. D. Haddock

**Affiliations:** 1 Sorbonne Université, CNRS, Laboratoire d’Océanographie de Villefranche, LOV, F-06230 Villefranche-sur-mer, France; 2 Monterey Bay Aquarium Research Institute (MBARI), 7700 Sandholdt Road, Moss Landing, 95039 CA USA; 3 Marine Biology Laboratory, Earth and Life Institute, Université catholique de Louvain, 3 place croix du sud, 1348 Louvain-La-Neuve, Belgium

**Keywords:** Biodiversity, Ecology

## Abstract

Bioluminescence is a prominent functional trait used for visual communication. A recent quantification showed that in pelagic ecosystems more than 75% of individual macro-planktonic organisms are categorized as able to emit light. In benthic ecosystems, only a few censuses have been done, and were based on a limited number of observations. In this study, our dataset is based on observations from remotely operated vehicle (ROV) dives conducted from 1991–2016, spanning 0–3,972 m depth. Data were collected in the greater Monterey Bay area in central California, USA and include 369,326 pelagic and 154,275 epibenthic observations at Davidson Seamount, Guide Seamount, Sur Ridge and Monterey Bay. Because direct observation of *in situ* bioluminescence remains a technical challenge, taxa from ROV observations were categorized based on knowledge gained from the literature to assess bioluminescence status. We found that between 30–41% of the individual observed benthic organisms were categorized as capable of emitting light, with a strong difference between benthic and pelagic ecosystems. We conclude that overall variability in the distribution of bioluminescent organisms is related to the major differences between benthic and pelagic habitats in the deep ocean. This study may serve as the basis of future investigations linking the optical properties of various habitats and the variability of bioluminescent organism distributions.

## Introduction

In the ocean, the largest ecosystem on earth, light emission by organisms is one the most effective ways to communicate, due to the transparency and optical homogeneity of the midwater environment. Bioluminescence allows for finding mates, escaping predators, or attracting prey^1^, and organisms can also use bioluminescence for multiple roles. However, due to their fragility and the challenges of accessing the deep ocean, bioluminescent organisms are difficult to observe alive or *in situ*. Most of the uses of this capability remain hypotheses, based on animal’s ecology and descriptions of bioluminescence characteristics of emission (wavelength, intensity, appearance, chemistry)^2^. Because of these ecological functions, bioluminescence capability has been described as a defining trait^3^. Traits of organisms are characteristics linked to morphology, physiology, life cycle, or behavior^4,5^ that affect the individual fitness of organisms. They give clues to interactions occurring at the community level, within trophic networks as well as between organisms and their environment^4^. Because community ecologists have thoroughly studied species diversity, we now have a better understanding of how the environment shapes species composition in benthic systems^6–8^. However, we know relatively little about how environmental variability relates to ecosystem functioning. A trait-based approach to assess the similarity of organisms based on functional and morphological traits is becoming more relevant to address general ecological rules in community ecology. The visual systems of many midwater and benthic (seafloor) organisms, like fishes^9^, cephalopods, molluscs^10^, crustaceans^11^, decapods^12^ and even sea stars^13^, can be well developed and central to their behavior^14^. Other deep-living animals have been described as photosensitive^15,16^. For these visual organisms living below a few hundred meters depth, the main source of light is dim bioluminescent emission, which highlights the importance of this trait. In shallow neritic (coastal pelagic) and benthic environments, where the sun penetrates, only 1–2% of species have been estimated to be bioluminescent^17^. However, more than 75% of macro-plankton living in the water column have the ability to emit light^18^.

The benthic region exhibits different optical, physical, and biogeochemical properties than the mesopelagic habitat. Indeed, in contrast to the three-dimensional water column, the sea floor is primarily a two-dimensional environment. More than 90% of the deep-sea floor is composed of silt and clay^19^ with a potential overlying nepheloid layer and low visibility due to sediment re-suspension by currents and other disturbances. A main characteristic of deep sea benthic ecosystems is energy limitation, as benthic production strongly depends on detrital organic material produced by primary production in the euphotic zone of the ocean, then attenuated and remineralized in the water column above. Benthic fauna play pivotal roles in sedimentary organic matter diagenesis, nutrient cycling, and ecosystem functioning in the deep sea. Most benthic organisms are suspension, filter-feeders, and detritivores. But the deep sea floor is also characterized by temporal and spatial heterogeneity. While mostly covered in soft sediment, there are some widely spaced seamounts, hydrothermal vents, canyons, and whale falls. These island habitats result in patches of higher-biomass communities and possibly physical and bathymetric obstacles for light communication. In areas with physical obstacles, investing energy into long-distance communication is less effective than in homogeneous environments. Bioluminescence capability in benthic organisms is thought to be scarce due to the frequent incidental impacts of plankton and mechanical stimulation from currents and other benthic organisms roaming the seafloor. Interactions involving frequent bioluminescence emission would render communication less effective and require a high energetic investment^20^.

Reduced bioluminescent capability in benthic animals has been hypothesized^20^ based on *in situ* low-light cameras images and sampled organisms that were tested for bioluminescence. From these observations, about 20 of 100 sampled organisms were described as bioluminescent. In addition, the light emission for benthic animals occurred at longer wavelengths (460–520 nm) than that of mesopelagic organisms (440–500 nm)^21^.

Previous studies on the distribution of bioluminescence have shown differences between organisms’ habitats^17,20,21^. However, these studies have been based on a limited number of sampled organisms. Major challenges to sampling come from a restricted number of stations sampled, short duration of cruises, the escape behavior of animals, the limited capability of ROVs to catch and store organisms, and the lack of low-light cameras to document the physiological state of animals shipboard. For these reasons, the quantification of this capability based on direct observations remains challenging.

In this work, we compiled pelagic and benthic deep-sea data, based on visual observations of organisms seen by the Monterey Bay Aquarium Research Institute (MBARI)’s ROVs in the Monterey Bay area. The dataset covers animals observed in the water column and on the deep seafloor in the Monterey Canyon, Guide and Davidson Seamounts as well as Sur Ridge, between 0–3,972 m depth. We categorized this information with a database we created from the literature and direct scientific observations to assign bioluminescence ability to over 1,157 taxa. The objectives were to assess: (i) the extent of bioluminescence ability in deep-sea epibenthic organisms; (ii) whether bioluminescent organisms have a different distribution in pelagic vs. benthic ecosystems; or (iii) between various specific benthic ecosystems, (iv) depth-related spatial variability in bioluminescence use; (v) the gaps in our knowledge of bioluminescence capability in benthic organisms, highlighting the taxa that have not been tested in order to focus further investigations. This study takes trait-based approach to quantifying the occurrence of bioluminescence in the deep-sea benthos and documents the differences in the use of this trait at an ecosystem level.

## Dataset and Methods

### Site location and dataset description

**Figure 1:**
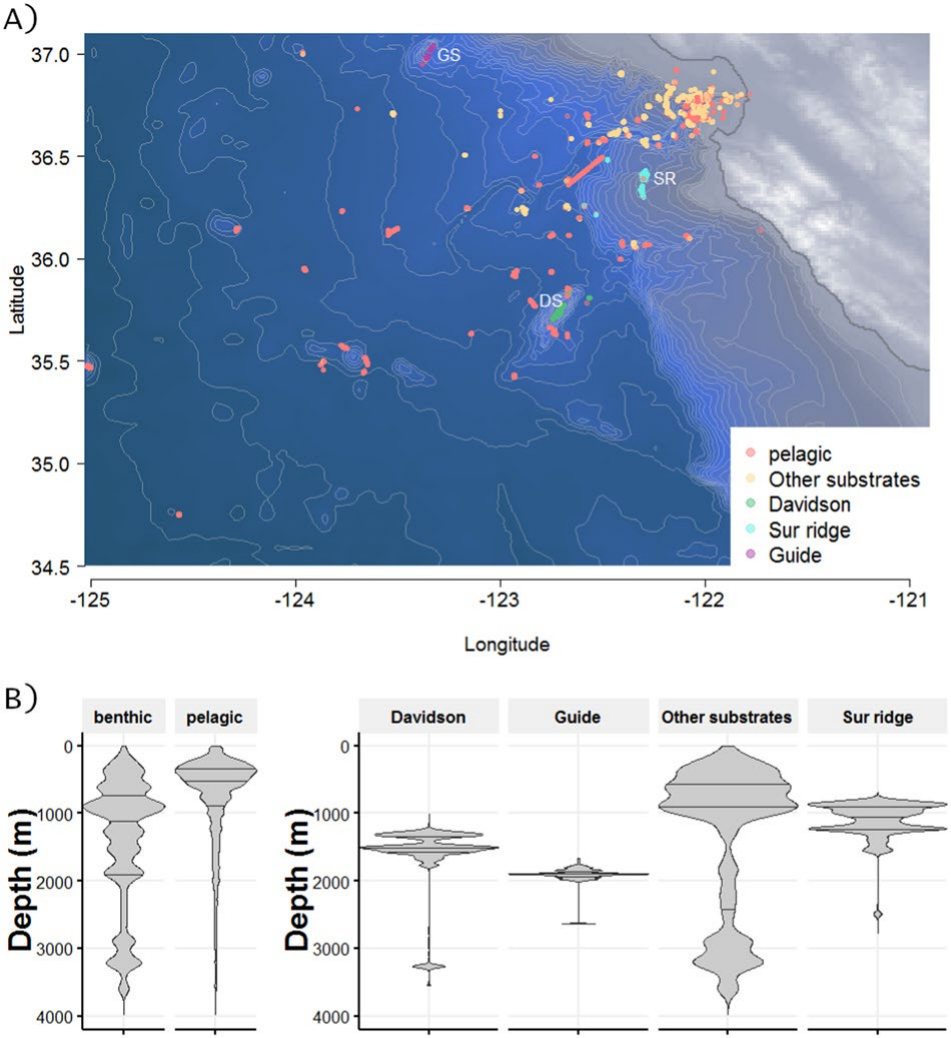
(**A**) Locations of the sampling sites for benthic and pelagic data sets. Samples are based on observations during ROV-dives off the California coast from 1991–2016. Videos were recorded, then analyzed using the Video Annotation Reference System (VARS) developed at MBARI, providing a large database of observations. DS: Davidson Seamount, GS: Guide Seamount, SR: Sur Ridge. The map is based on NOAA bathymetry (https://maps.ngdc.noaa.gov/viewers/bathymetry/), and sampling stations have been represented using R software. (**B**) Violin plots representing the distribution of the individuals observed over depths. On the left, total benthic and pelagic habitats are represented. On the right, benthic subhabitat categories are detailed for Davidson Seamount, Guide Seamount, Sur Ridge and Other substrates. The lines of the violin plots represent quantiles at 0.25, 0.5 and 0.75. There were 369,326 pelagic observations, and 154,275 benthic observations (17,906 at Davidson Seamount, 12,534 at Guide Seamount, 19,479 at Sur Ridge and 104,356 from Other Substrates).

Observations of benthic animals were documented MBARI’s remotely operated vehicles (ROV) on 621 dives between 1991–2016. Dives took place off the coast of California (Fig. 1), from nearshore waters to about 300 km offshore, (latitude from 34.28° to 37.04°N and longitude from 125.02° to 121.73°W). The sampled zone covers the continental shelf and slope, a major deep canyon (Monterey Canyon), and a fan valley down to 3,972 m depth (Fig. 1). Two regional offshore seamounts and one ridge were also included in the study. These three features rise above the deep-sea floor and are mainly composed of hard substrates. All sample locations except Guide Seamount are located within the Monterey Bay National Marine Sanctuary. Davidson Seamount (latitude 35.71°N, longitude 121.75°W) is located 121 km from the coast and measures about 42 km by 13.5 km. Its shallowest point is at 1,246 m with a base depth of 3,656 m. Guide Seamount (latitude 37.01°N, longitude 123.21°W) rises about 1,440 m above the seafloor and the peak sits at depth of 1,682 m. Both Davidson and Guide Seamounts are oriented on a NE/SW axis. Sur Ridge (latitude 36.21°N and longitude 122.18°W) is located 45 km west of Point Sur; it lies on a N–S axis at a maximum depth of 1350 m rising to 790 m. Pelagic records reflect data from the water column over offshore of Monterey Canyon and are available in Martini & Haddock^18^. Additional pelagic observations from the water column during benthic ROV dives descents and ascents were combined with the original dataset.

Dives were conducted between 6:00 and 19:00 local time. Over the period of this study, several ROVs were used (Tiburon, Doc Ricketts, Ventana) and four cameras were in use (Panasonic 3-chip, Sony 3-chip, Ikegami HDL40 and Sony HDTV). The focus distance was defined as 1.5 m from the camera. The typical volume observed by the camera in the water column varied between 1.2 and 3 m^3^ during this period. For benthic surveys, the field of view varied from 1–4 m^2^. All visible fauna in ROV videos (generally > 1 cm) were annotated by biologists using the MBARI’s open source Video Annotation Reference System (VARS)^22^ for database entry. Each observation of an animal was logged as a taxonomic entity, defined at its most specific taxonomic level observable from video, along with concurrent physical parameters (depth, latitude, longitude) and dive metadata (dive number, date). Data treatment was performed using Python scripts for VARS database retrieval, and R version 3.4.3^23^ for stats and plotting.

In order to deal with the assignment of bioluminescent categories for organisms on various taxonomic levels and to highlight more general patterns, taxa were grouped to higher taxonomic levels based on functional groups in the ecosystem. For example, in this study Hydroidolina mainly represents benthic hydroids, while pelagic Hydromedusae and Siphonophora were treated separately. A total of 29 groups were described as follow: Appendicularia, Chaetognatha, Hydromedusae, Scyphozoa, Thaliacea, Ctenophora, Rhizaria, Platyhelminthes, Bryozoa, Brachiopoda, Siphonophora, Nemertea, Crustacea, other Arthropoda, Asterozoa, Crinoidea, Hemichordata, Hexacorallia, Holothuroidea, Hydroidolina, Octocorallia, Echinoidea, Pteropoda, Cephalopoda, other Mollusca, Porifera, Ascidiacea, Annelida and Fishes (elasmobranchs, bony fish, Actinopterygii). Also, Holothuroids and Platyhelminthes may frequently swim and can be considered both pelagic and benthic, but we considered the benthos to be their primary habitat.

### Habitat and bioluminescence-trait attribution

All taxa were classified by habitat (pelagic, benthic) based on our knowledge and documentation of the seafloor where organisms occur. Subhabitat benthic categories were defined as: Davidson Seamount, Guide Seamount, Sur Ridge, (mainly hard substrates) and Other Substrates, which comprises mostly soft sediment habitats (Fig. 1, Table S1).

Following the methodology described in Martini and Haddock^18^, we assigned taxa to a bioluminescence category. Bioluminescent capability was classified into one of the following five categories: bioluminescent, likely bioluminescent, undefined, unlikely bioluminescent, and non-bioluminescent (Table 1). These descriptions are mainly based on previous literature^1,24–26^ and supplemented with additional unpublished observations since. As part of this study, some organisms have been assessed via direct observation using the following protocol. Organisms were gently captured using the ROV, either by suction samplers or by using sampling containers dedicated to biological collection. Once shipboard, the animals were placed in a completely dark and cold room. Observations were done by experts and trained observers to avoid mistakes due to light refraction, fluorescence or contamination.

(i) Animals were cleaned of the presence of other organisms, particles or mucus to avoid artefacts.
(ii) They were kept in separate containers, isolated in a cold dark room to rest for at least 10 minutes without any mechanical or light stimulation. Bioluminescence being a one-time reaction, animals need time to recover their bioluminescence capability after stimulation during sampling.
(iii) Observers acclimated themselves inside the dark room for a few minutes.
(iv) Animals were gently mechanically stimulated to see if bioluminescence was observed
(v) Mechanical stimulation was repeated to confirm observations.
(vi) As a positive control, organisms known to be bioluminescent were tested for comparison. Positive bioluminescence as well as negative observations were reported.

**Table 1:** Bioluminescence classification, from Martini and Haddock^18^.

Bioluminescent	Organisms described as bioluminescent in the literature or direct observations.
Likely	Organisms probably bioluminescent based on taxonomic assignment.
Non-bioluminescent	Organisms described as non-bioluminescent in the literature.
Unlikely	Organisms probably non-bioluminescent based on taxonomy assignment and direct observations.
Undefined	(1) Organisms that could not be categorized because they were equally likely to be bioluminescent or not, as well (2) organisms whose bioluminescent capability has not been examined or reported.

### Statistical test

A χ^2^-test (using function chisq.test() in R 3.4) was performed on a contingency table of observations for benthic, pelagic, Davidson and Guide Seamounts, and Sur Ridge habitats, defined as independent, and with a number above 5. The Null Hypothesis was the similarity between two habitats (Table 2).

**Table 2:** P-values for Chi-2 statistical test between ecosystems. Isolated hard substrates includes both seamounts and Sur Ridge.

	Pelagic	Other benthic substrates	Davidson Seamount	Guide Seamount	Sur Ridge	Benthic (all)	Isolated hard substrates
Pelagic		<0.01	<0.01	<0.01	<0.01	<0.01	<0.01
Other benthic substrates	<0.01		<0.01	<0.01	<0.01	<0.01	<0.01
Davidson Seamount	<0.01	<0.01		<0.01	<0.01	<0.01	<0.01
Guide Seamount	<0.01	<0.01	<0.01		0.04	0.06	<0.01
Sur Ridge	<0.01	<0.01	<0.01	0.04		<0.01	<0.01
Benthic (all)	<0.01	<0.01	<0.01	0.06	<0.01		<0.01
Isolated hard substrates	<0.01	<0.01	<0.01	<0.01	<0.01	<0.01	

### Ethical statements

Field operations were conducted under permit SC-4029 issued to SHD Haddock by the California Department of Fish and Wildlife, and through institutional permit # MBNMS-2005-002 from the Monterey Bay National Marine Sanctuary. For this study, specimens were only observed and not collected. Species used are unprotected and unregulated, and no vertebrates or octopus were used, so the International ethics guidelines are not invoked.

## Results

### Taxonomic observations and trait distribution over ecosystems

A total of 621 dives are included in this survey from years 1991–2016. In total 523,601 individual observations were included from among 29 taxa. There were 369,326 pelagic organisms from 599 dives (including the pelagic descent and ascent of some benthic dives) and 154,275 benthic individuals. Benthic subhabitats included: Davidson Seamount (n = 17,906; 28 dives), Guide Seamount (n = 12,534; 3 dives) and Sur Ridge (n = 19,479; 27 dives) and Other Substrates (n = 104,356; 523 dives) (Fig. 1).

**Figure 2:**
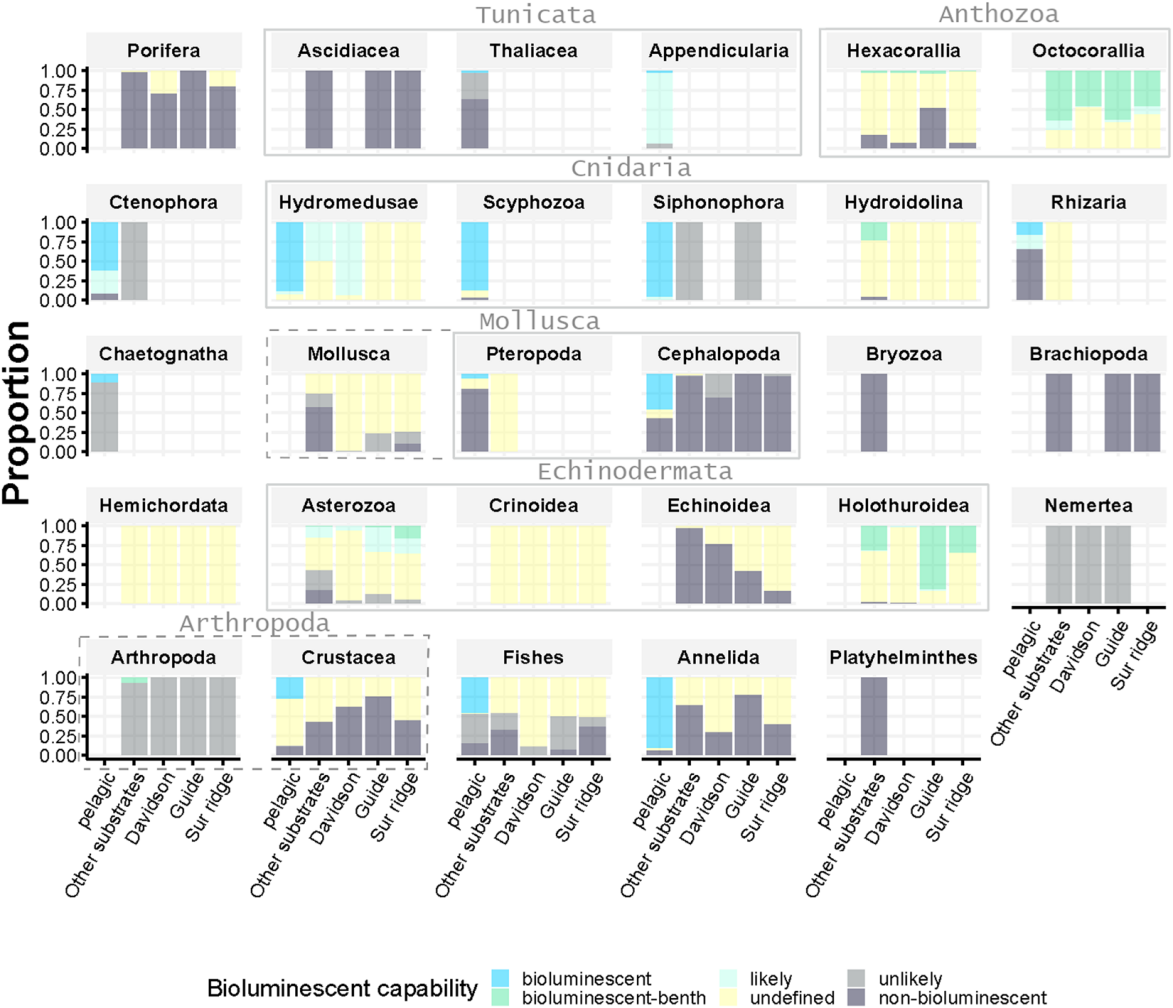
Proportion of total bioluminescence capability (y-axis) for each of the 29 taxa observed in the dataset (individual panels), and divided between subhabitats (pelagic, Other substrates, Davidson Seamount, Guide Seamount, and Sur Ridge, x-axis). Grey bounding boxes around panels show related taxonomic groups: Tunicata (Ascidiacea, Thaliacea and Appendicularia), Anthozoa (Hexacorallia and Octocorallia), Cnidaria (Hydromedusae, Scyphozoa, Siphonophora and other Hydroidolina), Mollusca (Pteropoda and Cephalopoda and other Mollusca), Echinodermata (Asterozoa, Crinoidea, Echinoidea, and Holothuroidea) and Arthropoda (Crustacea and other Arthropoda).

The proportion of bioluminescent capability of each of the 29 taxa observed in the dataset was described within each habitat (pelagic and 4 benthic subhabitats), (Fig. 2). Taxa observed exclusively in pelagic environments included Appendicularia (14.5%), Chaetognatha (11.1%), and Thaliacea. Thaliacea, Rhizaria, Cephalopoda, Scyphozoa and Pteropoda were each less than 5% of pelagic observations (Table S1). Appendicularia, Hydromedusae and Scyphozoa are mostly bioluminescent (93.8, 100.0 and 96.6% of bioluminescent/likely bioluminescent, respectively, Fig. 2) while Chaetognatha, Thaliacea and Pteropoda are mainly non-bioluminescent (11.2, 2.5 and 6.4% of bioluminescent/likely bioluminescent, respectively, Fig. 2).

Taxa observed exclusively in benthic environments included Octocorallia (15.2%), Asterozoa (11.1%), Holothuroidea (9.8%), Hexacorallia (9.1%), Porifera (9.1%), Crinoidea (7.0%), Echinoidea (5.3%) and Mollusca (3.3%) (Table S1). Ascidiacea, Brachiopoda, Bryozoa, Echinoidea, Porifera, and Nemertea are 100% non-bioluminescent/unlikely, while, Holothuroidea and Octocorallia appear to be 94.3% and 100% bioluminescent/likely. Octocorallia, specifically sea pens and bamboo corals, as well as brittle stars were highly bioluminescent. However these high percentages only rely on defined status, and around 66.1%, and 36.3% of Holothuroidea and Octocorallia, respectively, remain undefined. Some other benthic taxa fall strongly into the undefined (unknown status) bioluminescent category, including Hydroidolina (77.2% undefined), Hexacorallia (80.7% undefined) and Crinoidea, Hemichordata, Pteropoda, and Rhizaria (almost 100% undefined).

This trait is not exclusively present or absent within benthic taxonomic categories (Fig. 2). Within Asterozoa: *Benthopecten*, *Hymenaster koehleri* (seastars), and *Ophiomusium*, and *Ophiacantha* (brittle stars) are known to be bioluminescent^27,28^. *Stylasteridae* is a non-bioluminescent Hydroidolina, while most other taxa are bioluminescent. Nemertea, Echinoidea, Ascidiacea and Mollusca (excluding Pteropoda and Cephalopoda) were all undefined, non-bioluminescent or unlikely bioluminescent.

**Figure 3:**
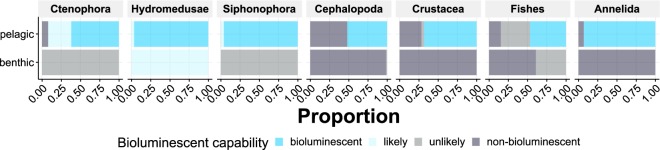
Distribution of bioluminescence capability among individuals in taxa present in both benthic and pelagic ecosystems. Numbers of observations are indicated as N = (numbers for benthic)/N = (number for pelagic). Cephalopoda (N = 985/N = 4,993), Ctenophora (N = 234/N = 28,268), Hydromedusae (N = 4,077/N = 64,500), Siphonophora (N = 3/N = 54,703), Crustacea (N = 8,345/N = 40,608), Annelida (N = 2,401/N = 35,409), Fishes (N = 28,995/N = 16,156).

While comparing the seven taxa (Cephalopoda, Ctenophora, Hydromedusae, Siphonophora, Crustacea, Annelida and Fishes, Fig. 3) observed in both pelagic and benthic ecosystems, we found that pelagic Annelida, Ctenophora and Siphonophora are more than 90% bioluminescent/likely, while none of the benthic taxa are known to exhibit this trait. One exception is Hydromedusae with about 100% benthic likely bioluminescent observations, represented by *Benthocodon* and *Ptychogastria*. For Cephalopoda, Fishes, and Crustacea, fewer pelagic individuals exhibit the bioluminescent/likely bioluminescent trait (51.8, 46.6 and 68.2% respectively) and only very rare (5 observations) of Fishes (Lophiiformes and *Porichthys*) were bioluminescent in benthic ecosystems. Benthic siphonophores were only represented by a single taxa *Dromalia alexandri* (unlikely bioluminescent) with just three observations in this data set. Benthic Cephalopoda included *Octopus*, *Benthoctopus*, *Graneledone boreopacifica*, and *Enteroctopus dofleini*. Benthic Crustacea included *Chionoecetes tanneri*, *Munidopsis*, *Paralomis*, *Storthyngura pulchra*, and *Pagurus tanneri*. Benthic Ctenophora is comprised only of two undescribed platyctenes, which have been found to be non-luminous in testing.

**Figure 4:**
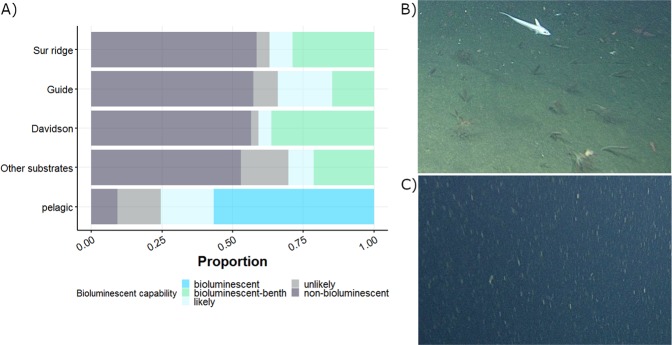
(**A**) Quantification of bioluminescence among various benthic subhabitats and the pelagic ecosystem. There were 154, 275 total benthic observations (19,479 at Sur Ridge, 12,534 at Guide Seamount, 17,906 at Davidson Seamount, and 104,356 from Other Substrates), as well as 369, 326 pelagic observations. (**B**) Benthic ecosystem, photo taken at Davidson Seamount by ROV, (**C**) Pelagic ecosystem, photos taken in the water column by ROV.

Analysis for this dataset shows that benthic ecosystems support a significantly smaller percentage of bioluminescent animals compared to pelagic ecosystems (32.4% vs. 75.5%, p-value < 0.01, Table 2). Comparisons between the benthic subhabitats (Fig. 4A) shows that Sur Ridge (36.1% bioluminescent), Guide Seamount (34.0%), Davidson Seamount (40.9%) and other substrates (30.4%) are statistically different one from each other (Chi-squared test with p-values < 0.01).

### Depth and spatial quantification of bioluminescence between ecosystems

**Figure 5:**
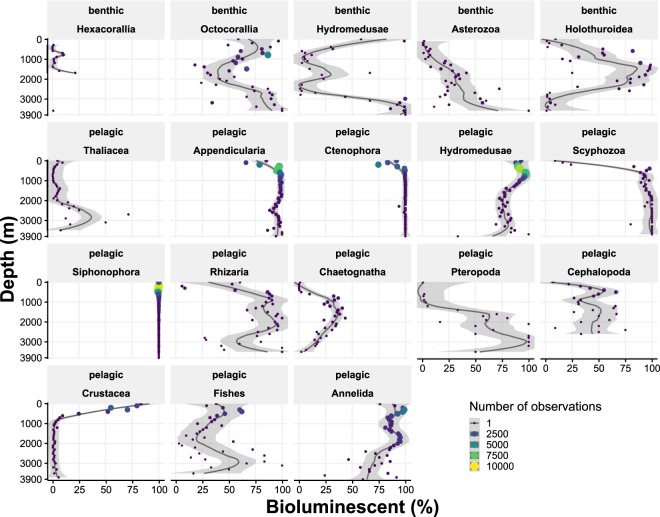
Profiles of the percentage of bioluminescent and likely-bioluminescent individuals per group and by depth. Only taxa observed at more than 4 different depths have been represented (benthic Arthropoda, Fishes and Hydroidolina have been removed). The black line represents a local loess regression (span = 0.5) with the grey bands being the 0.95 confidence interval around the smooth fit. The size and color scale show the maximum number of observations per class, used to weight the regression.

Pelagic observations show an increase in the percentage of bioluminescent individuals for some taxa (Appendicularia, Chaetognatha, Scyphozoa) and a decrease for other groups (Crustacea), below 700 m depth (Fig. 5). Bioluminescent Holothuroidea increase between 1,000 and 2,000 m depth (reaching 98.6% at 1,350 m) while the percent of Octocorallia decreased with an opposite pattern, with a minimum around 1,350 m depth (reaching 18.0%). Benthic Arthropoda, Fishes, and Hydroidolina, are not represented because they have been observed at only few (less than 4) different depths (Fig. 5).

**Figure 6:**
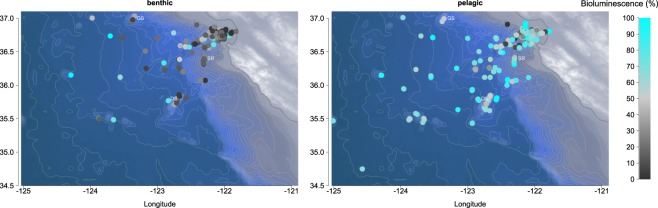
Spatial variability in bioluminescent organisms in and near Monterey Bay, CA. The map is based on NOAA bathymetry (https://maps.ngdc.noaa.gov/viewers/bathymetry/), and bioluminescence percentages have been represented using R software.

Overall the spatial distribution of pelagic bioluminescent organisms within the Monterey Bay area (Fig. 6) shows no clear pattern in distance from the shore.

### Quantification of bioluminescence between ecosystems over time

**Figure 7:**
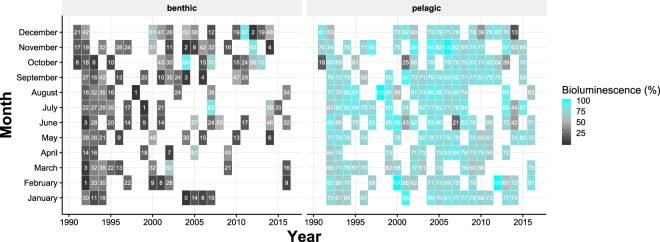
Variability of the percentage of bioluminescent and likely bioluminescent organisms over months and years (undefined are not taken into account). This variability includes spatial differences within the sampling stations and only pelagic and benthic data have been represented due to the lower sampling rate of Davidson Seamount, Guide Seamount and Sur Ridge.

These data are opportunistic and non-quantitative in both time and space due to the highly varied scientific focus of ROV cruises. These limitations make the data set unsuitable for time-series and seasonality analyses (Fig. 7).

## Discussion

While quantification of bioluminescence between ecosystems has been previously investigated based on a limited number of *in situ* opportunities, this study proposes a trait-based methodology to quantify differences in the diversity of bioluminescence between pelagic and benthic ecosystems. Based on a large dataset and extensive knowledge from the literature, we show that the ability to bioluminesce may be strongly driven by habitat. We hypothesize that this is probably related to differences in optical properties between pelagic and benthic environments (Fig. 4). While more than 75% of individual pelagic taxa are known to emit light, in the various four benthic ecosystems we studied, only 30–41% of individuals have this ability.

### Sampling limitations and biases

In order to describe the use of a trait in organisms, such as bioluminescence, two different approaches can be employed. The first approach is to directly document the characteristic for each individual sampled or observed. For bioluminescence, the opportunity to observe light emission *in situ* is currently a large challenge. This ability is strongly related to the physiological state of organisms and is triggered by mechanical or light stimulation. If the specimen is stimulated during collection, it will need some time to rest before being able to re-emit light. Among the sampling methods in oceanography, nets are damaging to fragile creatures and stimulate bioluminescence emission, so most organisms cannot recover after rough manipulation. *In situ* scuba-diving is one traditional way to capture and observe animals but has a very limited sampling-depth. Imagery is an effective and reproducible tool to describe marine ecosystems with qualitative and quantitative data after footage or still images are annotated by experts^29^. Low-light cameras sensitive enough to detect the dim light of bioluminescence have only recently been developed and have been rarely implemented on ROVs, AUVs (autonomous underwater vehicles), or other long-term deployed platforms. For unmanned vehicles and cameras, emitting white light as well as low-light would be required in order to see and document which organisms are present. The second approach is to study potential traits^4,30^. In a previous study^18^, we merged knowledge from the literature describing organisms that exhibited the ability to bioluminesce with surveys describing planktonic organisms. The use of imaging data as the basis of individual observations considerably increases the amount of information, and consequently the relevance of quantitative conclusions. This method is only possible if sufficient information is available from the literature to describe this trait among deep-sea animals. In this work, such an approach is useful in order to have strong statistical sampling of the trait among a large number of organisms, some of which are rarely observed.

The use of ROV imagery as a tool for quantification has demonstrated caveats^18,31,32^. The attraction or repulsion of motile organisms sensitive to the light and sounds of the vehicle introduces bias in both pelagic and benthic environments. ROV sampling favors gelatinous animals over hard-bodied ones^33^ and are therefore better suited to counting gelatinous organisms, which are often underestimated by net sampling. In benthic environments fast, mobile animals like fishes may avoid the ROV, and remain uncounted. Nonetheless, ROVs are currently the most efficient tool giving access to a large number of deep-sea observations for more robust statistical interpretations.

Literature information can also be biased in two opposite ways. First, literature eventually favors more false-bioluminescent reports. For example, bioluminescence in Porifera has actually been reported in the literature^34,35^. However, few decades later, these observations have been reported as doubtful and more likely due to organisms, such as worms, annelids or other invertebrates living in the sponge tissue^36,37^. Then, direct observations can also miss light emission. Indeed, deep sea organisms are sampled from thousands of meters away from the surface and brought back on board, where they face not only pressure decrease but temperature and light increases that could affect organism’s responsiveness. When captured, animals are mechanically stimulated and potentially emit bioluminescence. A resting time of several minutes is needed for animal to recover their bioluminescence capability but many organisms cannot survive for long at the surface. In our study, we carefully crossed information available and made some direct observations, on several individuals, when possible to validate or invalidate the list of bioluminescent capability. Indeed, during MBARI cruises, animals sampled by ROVs were checked for bioluminescence, when possible. These direct-observations were not necessarily “new” ones (not listed in the literature) but an opportunity to validate some organisms in our reference list. Several Octocorallia, some Hexacorallia, Asterozoa, and Holothuroidea, as well as few Mollusca and Ophiuroidea have been checked for bioluminescence onboard.

In the water column, the environment is relatively homogeneous. The main source of variability is depth-related, due to the daytime sampling in this study, day/night vertical migration potentially influences the profiles of some pelagic organisms (such as crustaceans) in the water column (see^18^ for more information). Because of this relatively homogeneous environment, pelagic surveys are considered representative of the water column across broad regions. In the deep benthos, habitat heterogeneity is much higher and thus observations are very specific to an exact location visited. Sites such as whale-falls, seamounts, cold-seeps, and the abyssal plain all have depth-related and habitat-related biological communities. Such quantitative bias has been lowered by looking at the relative proportion of taxa and traits between some of these ecosystems (Figs 2 and 3). Here, three specific locations (Guide Seamount, Davidson Seamount, Sur Ridge) and wider benthic observations have been differentiated to test spatial variability within these ecosystems, as well as variability in depth. Taxonomic diversity was expected between seamounts, but due to the relatively proximity of Davidson, Guide and Sur Ridge, it has previously been stated that only low endemism was observed within such distances^38^. Moreover, a Chi-squared test shows that there are differences between almost all benthic sites (except benthic and Sur Ridge), supporting the need to study them independently, although the percentage of bioluminescent organisms in all those benthic stations was clearly different than the pelagic dataset (between 30–41% for benthic and 75% for pelagic). By taking into account those differences, we suggest that these observations are representative of benthic and pelagic fauna of greater Monterey deep water regions. Further studies would allow characterizing whether differences exist between oceanic regions.

In this study we also highlight the large proportion of organisms where bioluminescence capability remains undefined. This uncertainty includes some of the main taxa observed in the benthic environment. Deep-sea organisms are understudied and poorly known due to technical constraints and rare sampling opportunities. As an example, local species of Crinoidea and Hemichordates have not been characterized. The results of a recent expedition “Sampling the Abyss” revealed a percentage of about 49% luminous Echinoderm species. From this percentage more than a half were not known to be bioluminescent (J.M. pers. comm.). Interestingly, during this cruise, four species of Crinoidea within the Comatulida order (observed on the East coast of Australia, from Hobart to Brisbane but not present in Monterey Bay) were bioluminescent (J.M. pers. comm.). Some abundant species in the pelagic ecosystem, such as the chaetognaths *Eukrohnia fowleri* and *Caecosagitta macrocephala*, have only recently been discovered to be bioluminescent, while the related species are non-luminous^39,40^. Most Hydroidolina, Holothuroidea, and Octocorallia individuals observed and defined are bioluminescent or likely bioluminescent, but many species remain untested. Thus far, no Porifera or Echinoidea have been described as bioluminescent but the number of direct observations are very limited. To deal with this lack of direct *in situ* observations of bioluminescence emission, our approach has been to document current knowledge and identify areas where this research is lacking. More data at the species level will allow a more thorough assessment and quantification of this important morphological trait within benthic ecosystems and has already been of major importance in the pelagic environment.

### Ecology

While it has been shown that the capability of light emission by organisms is a widely distributed ecological trait for pelagic taxa, here we quantify its distribution for epibenthic fauna. Bioluminescence is known to strongly influence ecological relationships between animals through predator-prey relationships, and in finding a partner^1^. It has been hypothesized^17^ that there are more frequent and complex uses of bioluminescence in the pelagic environment compared to the benthic realm, due to limitations in its effectiveness. Obstacles like rocks, and water turbidity on the deep seafloor may block light emission, making this function too physiologically expensive. Representative images of benthic and pelagic ecosystems (Fig. 4B) illustrate the differences in such optical properties. Further investigations are needed to test the impact of seafloor rugosity, slope and aspect on the proportion of bioluminescent benthic organisms. In benthic ecosystems, deep-sea organisms are currently under increasing pressure due to anthropogenic stressors such as eutrophication^19^, deep-sea fishing, mineral mining, and oil and gas extraction^41^. One of the expectations arising from this study is that reductions in the clarity of the water column and in benthic ecosystems through human activities could have major effects on biodiversity. As more benthic organisms are studied and tested for light emission, the estimated potential impact of anthropogenic changes will surely increase.

Competition for resources is known to be a strong driver of communities where food is limited, with decreasing organic matter availability with depth^42^. One might expect strong differences between the two main ocean ecosystems with more diversified feeding strategies (including using light emission to attract food) in a vast 3-dimensional, environment compared to the 2-dimensional deep seafloor.

Relationships between organisms based on bioluminescence may be more complex than we know. There are examples of symbioses where a non-bioluminescent partner interacts with a bioluminescent host (and vice-versa). In Monterey Bay at locations sampled in this study, *Neolithodes diomedeae* (juvenile crab, non-bioluminescent) and *Scotoplanes* (bioluminescent) form a symbiotic association^43^ but the bioluminescence potential advantage remains unstudied. It may be that there is some indirect benefit for the non-bioluminescent partner, such as finding and settling on the host holothurian. Among benthic taxa, motile (Holothuroidea, Annelida, Fishes or Cephalopoda) as well as sessile (Octocorallia, Anemones) animals have been observed and described as bioluminescent. Some of them have a complex life cycle involving both pelagic and benthic phases. Very little is known about the role of bioluminescence during those different life stages and in-depth investigations may reveal new ecological functions for bioluminescence.

## Conclusion

There are still numerous discoveries to be made regarding the functioning of deep-sea organisms. For those organisms bioluminescence is integral to communication, finding food, and predator avoidance and a better understanding of this capability is of major interest for deep-sea ecology. In this study, we found that between 30–41% of the individual observed benthic organisms were capable of emitting light, with a strong difference between pelagic and benthic ecosystems. We also highlighted taxa in which more effort has to be pursued to increase our knowledge about bioluminescent capability. The derived values gained from this study could potentially be applied at other deep-sea sites in the world.

## Supplementary information

supplementary dataset
